# Risk assessment models for potential use in the emergency department have lower predictive ability in older patients compared to the middle-aged for short-term mortality – a retrospective cohort study

**DOI:** 10.1186/s12877-019-1154-7

**Published:** 2019-05-16

**Authors:** Martin Schultz, Line Jee Hartmann Rasmussen, Nicolas Carlson, Rasmus Bo Hasselbalch, Birgitte Nybo Jensen, Lotte Usinger, Jesper Eugen-Olsen, Christian Torp-Pedersen, Lars Simon Rasmussen, Kasper Karmark Iversen

**Affiliations:** 1 Department of Cardiology, Herlev and Gentofte Hospital, University of Copenhagen, Herlev Ringvej 75, DK-2730 Herlev, Denmark; 2 Department of Internal Medicine and Geriatrics, Herlev and Gentofte Hospital, University of Copenhagen, Herlev, Denmark; 3 Clinical Research Centre, Amager and Hvidovre Hospital, University of Copenhagen, Hvidovre, Denmark; 4 The Danish Heart Foundation, Copenhagen, Denmark; 5 Department of Emergency Medicine, Bispebjerg Hospital, University of Copenhagen, Copenhagen, Denmark; 6 Department of Health, Science and Technology, Aalborg University Hospital, Aalborg, Denmark; 7 Department of Cardiology and Epidemiology/Biostatistics, Aalborg University Hospital, Aalborg, Denmark; 8 Department of Anaesthesia, Centre of Head and Orthopaedics, Rigshospitalet, University of Copenhagen, Copenhagen, Denmark

**Keywords:** Emergency department, Older patients, Risk assessment, Triage

## Abstract

**Background:**

Older patients is a complex group at increased risk of adverse outcomes compared to younger patients, which should be considered in the risk assessment performed in emergency departments. We evaluated whether the predictive ability of different risk assessment models for acutely admitted patients is affected by age.

**Methods:**

Cohort study of middle-aged and older patients. We investigated the accuracy in discriminating between survivors and non-survivors within 7 days of different risk assessment models; a traditional triage algorithm, a triage algorithm with clinical assessment, vital signs, routine biomarkers, and the prognostic biomarker soluble urokinase plasminogen activator receptor (suPAR).

**Results:**

The cohort included 22,653 (53.2%) middle-aged patients (age 40–69 years), and 19,889 (46.8%) older patients (aged 70+ years). Death within 7 days occurred in 139 patients (0.6%) in middle-aged patients and 596 (3.0%) of the older patients. The models based on vital signs and routine biomarkers had the highest area under the curve (AUC), and both were significantly better at discriminating 7-day mortality in middle-aged patients compared to older patients; AUC (95% CI): 0.88 (0.84–0.91), 0.75 (0.72–0.78), *P* < 0.01, and 0.86 (0.82–0.90), 0.76 (0.73–0.78), *P* < 0.001. In a subgroup of the total cohort (6.400 patients, 15.0%), the suPAR level was available. suPAR had the highest AUC of all individual predictors with no significant difference between the age groups, but further research in this biomarker is required before it can be used.

**Conclusion:**

The predictive value was lower in older patients compared to middle-aged patients for all investigated models. Vital signs or routine biomarkers constituted the best models for predicting 7-day mortality and were better than the traditional triage model. Hence, the current risk assessment for short-term mortality can be strengthened, but modifications for age should be considered when constructing new risk assessment models in the emergency department.

**Electronic supplementary material:**

The online version of this article (10.1186/s12877-019-1154-7) contains supplementary material, which is available to authorized users.

## Background

Emergency departments (EDs) must prioritise treatment of patients of all ages and health status according to urgency while counterbalancing the negative effects of ED crowding on patient safety [1, 2]. EDs commonly employ triage algorithms to assess risk and prioritise according to the perceived urgency of patients’ conditions. These algorithms typically consist of a combination of vital signs and primary complaints for risk assessment [3].

Older patients aged 65 years and older account for 12–24% of all ED visits; additionally, both the number of visits and the utilisation of resources in the ED are increasing [4, 5]. Compared to younger patients, the older often have a greater burden of comorbidities, more severe illnesses [4, 6, 7], and often present with atypical symptoms and nonspecific complaints [4, 8, 9]. Therefore, older patients constitute a complex group at high risk; within 3 months following an ED visit 20% of older patients had been hospitalised, 20% revisited the ED, and approximately 5% were dead [5]. Additionally, age-related changes in physiology may result in reduced variability and decreased response to stress [10], rendering vital signs less useful for assessment of urgency and severity of acute illness in the older [10, 11]. However, none of the currently used triage algorithms stratifies patients according to age [12].

Risk assessment in the ED can be done using one of the traditional triage algorithms [12], but recent studies have attempted to improve risk assessment using different approaches or adding new elements. Incorporation clinical assessment is one possibility as physicians first clinical impression is associated with morbidity and mortality, and because ED staff using only clinical intuition can identify patients at risk of death [13, 14]. Different vital signs are associated with adverse outcome and using these in risk scores or simply the number of abnormal vital signs can identify patients at risk, providing a second approach for a better risk assessment [15, 16]. A third possibility, is risk assessment using the routine biomarkers analysed from blood samples during acute admission. Many of these biomarkers have value in risk stratification, in addition to their diagnostic value. For instance C-reactive protein (CRP) and albumin are associated with mortality, [17, 18] and a statistical model containing eight routine biomarkers has been shown to be significantly better in predicting mortality compared to traditional triage [19]. Finally, newly discovered experimental biomarkers have been found to carry prognostic information that allows for an accurate risk discrimination [20–22]. One of these prognostic biomarkers is soluble urokinase plasminogen activator receptor (suPAR), which is associated with disease severity of several acute and chronic conditions, and with length of stay, readmissions and mortality in ED patients [23–25].

The optimal and best approach, however, remains undetermined. The aim of this study was to evaluate the predictive ability of different models for risk assessment regarding short-term mortality in ED patients and to compare the accuracy of the models in middle-aged and older patients.

## Methods

### Study design

For this study of middle-aged and older patients presenting at an ED, we used data from the TRIAGE II and TRIAGE III studies [26, 27]. In brief, the TRIAGE II study was a prospective, interventional cluster-randomised trial comparing a newly developed triage algorithm, which adds a clinical assessment to a traditional triage algorithm [27]. The TRIAGE III study was a cluster-randomised interventional trial investigating the effect of introducing the nonspecific prognostic biomarker soluble urokinase plasminogen activator receptor (suPAR) as a routine blood test in the ED [26, 28].

### Setting

TRIAGE II and TRIAGE III included ED patients at Bispebjerg Hospital and Herlev Hospital, Denmark; both are University hospitals with 24-h acute care of patients, offering all internal medicine specialities, general and orthopaedic surgery, intensive care, and level-2 trauma care.

### Patients

Patients’ first ED visit from the TRIAGE studies was included, subsequent readmissions were excluded. Scientific research use different age cut-offs to define older patients ranging from 60 to 75 years [4], in the present study we classified patients aged 40 to 69 years as middle-aged, and patients aged 70+ years as older, in accordance with previous research from emergency departments [29–31]. Patients younger than 40 years were excluded. The TRIAGE studies included ED patients from the same locations in a continuous time period making the study populations similar. To assess the different risk assessment models, we combined the two study populations into one large cohort for this study.

### Data collection

Vital signs, triage categories, and results of blood tests were recorded at the ED visit. Vital signs, blood tests and triage categories were retrieved from local hospital databases. Follow-up data on hospital admissions, discharges, and diagnoses were obtained from the Danish National Patient Registry, [32, 33] and vital status at the end of each TRIAGE study was obtained from the Danish Civil Registration System, using patients’ unique Danish civil registration number.

### Outcome measures

Outcomes were all-cause mortality within two and 7 days.

### Risk assessment models

We assessed five different types of risk assessment models in the ED; I) a traditional triage algorithm, II) a triage algorithm with clinical assessment, III) vital signs, IV) routine biomarkers, and finally, V) the experimental biomarker suPAR, and compared the predictive abilities of middle-aged patients to older patients.

#### Traditional triage algorithm

The commonly used triage algorithm in Denmark is a local adaptation of “Adaptive Process Triage” (ADAPT), [34] which is based on vital signs and the presenting complaint [35, 36]. Patients are divided into five categories: Red (most urgent), Orange, Yellow, Green (least urgent), and Blue (minor injuries). In this analysis, patients triaged “Green” and “Blue” were combined. ADAPT is similar to other traditional 5-level triage algorithms [12].

#### Triage with clinical assessment

“The Copenhagen Triage algorithm” (CTA) is a newly developed triage algorithm with five categories (similar to ADAPT) based on measurement of vital signs and supplemental of oxygen, followed by a systematic clinical assessment by the ED staff, permitting modification of the final triage category [27].

#### Vital signs

The vital signs; heart rate, respiratory rate, arterial oxygen saturation, systolic blood pressure, and temperature were measured at arrival to the ED.

#### Routine biomarkers

In the present study, we included levels of albumin, CRP, creatinine, haemoglobin, leukocyte count, sodium, potassium, and platelets, as they are routinely measured during acute admissions and were available.

#### Experimental biomarker

In recent years, several new biomarkers with prognostic information have been discovered as promising risk assessment tools for use in the ED [37]. suPAR is a nonspecific biomarker of inflammation associated with mortality [21, 24].

### Statistics

Continuous variables are presented with median and interquartile range (IQR) or mean (standard deviation), and categorical variables as number (n) and percentages (%). Baseline characteristics were compared using the chi-square test, Student’s two-sample t-test, and Wilcoxon rank-sum test. The ability of triage, vital signs, and biomarkers to discriminate on mortality was assessed using the area under the curve (AUC) for receiver operating characteristics curves. AUCs were compared using the DeLong’s method [38]. Triage categories, vital signs, and biomarkers were used as continuous variables in logistic regression to calculate AUCs. Combined models were created using multiple logistic regression. Patients with missing predictors were not included in the respective analyses. A *P*-value below 0.05 was considered statistically significant. Statistical analyses were performed in R version 3.3.3 [39] using the pROC package [40].

## Results

### Study population

The TRIAGE II study included ED visits of 39,883 patients from March 1, 2015, to January 31, 2016, and the TRIAGE III study included ED visits of 16,801 patients from January 11, 2016, to June 6, 2016. After exclusion of patients younger than 40 years, the combined cohort consisted of 42,542 patients (75.1%). The mean (SD) age was 67.2 (14.9) years, and 21,880 (51.4%) were women. At inclusion, 22,653 (53.2%) were age 40–69 years (middle-aged), and 19,889 (46.8%) were aged 70+ years (older). Death from all causes within 7 days occurred in 139 patients (0.6%) in the group of middle-aged patients and in 596 (3.0%) in the group of older patients. All baseline characteristics were significantly different when comparing the two age-groups, except for temperature, Table 1. The two TRIAGE populations were comparable regarding age, sex, and outcomes, but minor, but statistically significant, differences in the levels of the biomarkers were observed (Additional file 1: Table S1).

**Table 1:** Baseline characteristics at first visit at the emergency department. Patients are grouped according to their age; 40–69 years (middle-aged) and 70+ years (older)

	Middle-aged *N* = 22,653	Older *N* = 19,889	*P*
Female sex, n (%)	10,687 (47.2)	11,193 (56.3)	< 0.001
Age (years), mean (SD)	55.5 (8.6)	80.7 (7.4)	
Triage: ADAPT	11,389 (50.3)	9746 (49.0)	
Triage category: Red, n (%)	418 (3.1)	482 (4.9)	< 0.001
Triage category: Orange, n (%)	3376 (24.0)	3038 (31.2)	0.02
Triage category: Yellow, n (%)	3953 (36.3)	3428 (35.1)	0.49
Triage category: Green, n (%)	3642 (36.5)	2798 (28.7)	< 0.001
Triage: CTA	7983 (35.2)	6655 (33.5)	
Triage category: Red, n (%)	189 (2.4)	225 (3.4)	< 0.001
Triage category: Orange, n (%)	1276 (16.0)	1143 (17.2)	0.05
Triage category: Yellow, n (%)	3092 (38.7)	2409 (36.2)	0.01
Triage category: Green, n (%)	3426 (42.9)	2878 (43.2)	0.70
Vital signs	13,200 (58.3)	10,992 (55.3)	
Heart rate (beats/min), mean (SD),	86 (20)	85 (20)	< 0.001
Arterial oxygen saturation (%), median (IQR)	97 (96–99)	96 (95–98)	< 0.001
Respiratory rate (breaths/min), mean (SD)	17 (3)	18 (4)	< 0.001
Systolic blood pressure (mmHg), mean (SD)	140 (25)	146 (29)	< 0.001
Temperature (°C), mean (SD)	36.8 (0.8)	36.8 (0.8)	0.07
Biomarkers levels, n (%)	13,032 (57.5)	13,039 (65.6)	
Albumin (g/L), median (IQR)	41 (37–47)	38 (34–41)	< 0.001
Creatinine (μmol/L), median (IQR)	72 (60–87)	83 (66–111)	< 0.001
CRP (mg/L), median (IQR)	4 (3–33)	11 (3–61)	< 0.001
Haemoglobin (mmol/L), median (IQR)	8.6 (7.8–9.2)	7.9 (7.0–8.6)	< 0.001
Leucocytes (× 10^9^/L), median (IQR)	8.6 (6.6–11.3)	8.9 (6.8–11.8)	< 0.001
Potassium (mmol/L), median (IQR),	3.9 (3.7–4.2)	4.0 (3.7–4.3)	< 0.001
Sodium (mmol/L), median (IQR)	139 (136–141)	138 (135–141)	< 0.001
Platelets (× 10^9^/L), median (IQR)	245 (200–300)	242 (195–309)	< 0.001
suPAR (ng/mL), median (IQR)	3.7 (2.8–5.2)	5.4 (4.0–7.5)	< 0.001
Mortality			
Mortality within 2-days, n (%)	73 (0.3)	263 (1.3)	< 0.001
Mortality within 7-days, n (%)	139 (0.6)	596 (3.0)	< 0.001

*CRP* C-reactive protein, *IQR* Interquartile range, *SD* Standard deviation, *suPAR* Soluble urokinase plasminogen activator receptor

### Predictive ability of different risk assessment models

AUCs for 2-day and 7-day mortality for all five risk assessment models stratified in age-groups are presented in Table 2, individual vital signs and biomarkers in Additional file 1: Table S2 and the AUCs regarding 7-day mortality is presented in Additional file 1: Figure S1.

**Table 2:** Comparison of AUCs in predicting short-term mortality of patients acutely presenting at the emergency department, grouped according to age: 40–69 years (middle-aged), and 70+ years (older)

AUC, 95% CI	Middle-aged *N* = 22,653	Older *N* = 19,889	*P*
ADAPT, 2-day mortality	0.80 (0.73–0.87)	0.76 (0.72–0.81)	0.40
ADAPT, 7-day mortality	0.72 (0.66–0.78)	0.71 (0.67–0.74)	0.69
CTA, 2-day mortality	0.83 (0.77–0.89)	0.78 (0.73–0.84)	0.22
CTA, 7-day mortality	0.79 (0.73–0.85)	0.73 (0.69–0.76)	0.09
Vital signs, 2-day mortality	0.89 (0.84–0.94)	0.81 (0.77–0.86)	0.02
Vital signs, 7-day mortality	0.88 (0.84–0.91)	0.75 (0.72–0.78)	< 0.001
Biomarkers, 2-day mortality	0.84 (0.78–0.90)	0.75 (0.71–0.79)	0.02
Biomarkers, 7-day mortality	0.86 (0.82–0.89)	0.76 (0.73–0.78)	< 0.001
suPAR, 2-day mortality	0.82 (0.66–0.97)	0.73 (0.64–0.81)	0.32
suPAR, 7-day mortality	0.82 (0.73–0.91)	0.77 (0.72–0.82)	0.32

*ADAPT* Adaptive process triage, *AUC* Area under the curve, *CI* Confidence interval, *CRP* C-reactive protein, *CTA* Copenhagen triage algorithm, *suPAR* Soluble urokinase plasminogen activator receptor, Vitals: Prediction model using four vital signs (hear rate, oxygen saturation, respiratory rate, systolic blood pressure), Biomarker: Predictive model using levels of seven routine biomarkers (albumin, creatinine, c-reactive protein, haemoglobin, leucocytes, potassium, sodium)

#### Traditional triage algorithm

ADAPT triage categories were recorded for 11,389 (50.3%) of middle-aged patients and 9746 (49.0%) of older patients. Significantly fewer middle-aged patients were triaged “Red” compared to the older, while more were triaged “Green” (Table 1). No difference in the predictive ability of short-term, all-cause mortality was observed between the two groups (Table 2):

#### Triage with clinical assessment

CTA triage categories were recorded for 7983 (35.2%) of middle-aged patients and 6655 (33.5%) of older patients. Comparison of the distribution in triage categories showed significantly more middle-aged patients in the “Yellow” category and less in the “Red” (Table 1). AUC analyses showed better prognostic ability compared to ADAPT, and no difference in the prognostic ability between age groups (Table 2).

#### Vital signs

A complete set of the five included vital signs was recorded in 13,200 (58.3%) of middle-aged- and 10,992 (55.3%) of older patients. Of five available vital signs, four had a predictive value of 2-day and 7-day mortality (not temperature). Of the remaining vital signs, the respiratory rate had the highest AUC and was significantly better at predicting short-term mortality among the middle-aged than among the older (Additional file 1: Table S2). Combining the vital signs (heart rate, arterial oxygen saturation, systolic blood pressure, and respiratory rate) in a prediction model showed significantly better predictive ability for short-term mortality among the middle-aged compared to the older (Table 2).

#### Routine biomarkers

Included routine biomarkers measured at ED visit were available in 13,032 (57.5%) of the middle-aged and 13,039 (65.6%) of the older. All routine biomarkers, except for platelets, had an individual predictive value of 2-day and 7-day mortality in both groups. Levels of albumin, CRP, haemoglobin, and sodium were significantly better at predicting short-term mortality in the middle-aged patients (Additional file 1: Table S2). A combined model consisting of seven biomarkers (platelets not included) had significantly higher AUC in predicting short-term mortality in the group of middle-aged patients compared to the older (Table 2).

#### Experimental biomarker

suPAR was available in 3227 (14.2%) middle-aged- and 3173 (16.0%) of the older patients. Its predictive ability was higher than individual vital signs or routine biomarkers and comparable to the triage algorithm (Additional file 1: Table S2). There was no difference observed among age-groups (Table 2).

### Overall predictive ability across age deciles

**Fig. 1:**
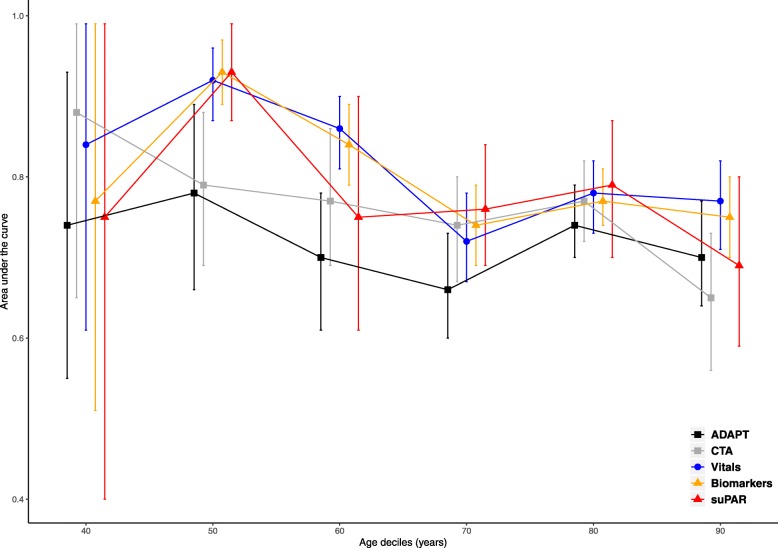
Area under the Curve for Receiver operating characteristics for all-cause mortality within 7 days for acutely admitted patients. The graph presents five different approaches to risk assess patients acutely presenting at the emergency department. Patients are stratified in age deciles according to their age at the first visit. The five approaches include; Two different triage algorithms; Adaptive Process Triage (ADAPT) and Copenhagen Triage Algorithm (CTA), a predictive model using four vital signs (heart rate, arterial oxygen saturation, respiratory rate and systolic blood pressure), a predictive model using levels of seven routine biomarkers (albumin, creatinine, c-reactive protein, haemoglobin, leucocytes, potassium, sodium), and the experimental biomarker soluble urokinase plasminogen activator receptor (suPAR). Mortality in age deciles; 40: 0.2%, 50: 0.4%, 60: 1.0%, 70: 1.9%, 80: 3.6%, 90: 5.2%

All-cause mortality within 7 days was increasing with increasing age. Stratifying the study population according to age deciles decreased the accuracy in all five risk assessment models, Fig. 1. In the two triage algorithms, this was not significant. The AUCs were significantly lower in the models consisting of vital signs, biomarkers, and suPAR when comparing age 50 and age 80 years, *p* < 0.001.

## Discussion

In this study of 42,452 acutely presenting patients, we found that risk assessment models based on vital signs and routine biomarkers, had the best predictive abilities compared to the other risk assessment models tested; both models were significantly better at discriminating on short-term mortality in middle-aged patients compared to older patients. Furthermore, all five risk assessment models included in this study showed fair to good abilities in discriminating between survivors and non-survivors at 7 days, but lower AUCs for the older.

The traditional triage algorithm (ADAPT), which is commonly used, was less accurate than the other models but did not differ significantly between age groups. Most of the included vital signs and biomarkers had individual predictive value but combining them in models yielded the highest accuracy. Among the individual routine biomarkers, albumin and CRP had the highest AUCs and performed significantly better in the middle-aged patients than in older patients. The experimental biomarker, suPAR, had the highest AUC of all investigated individual predictors, and there was no significant difference in the predictive value between the age groups. However, suPAR was only available in 14.2% of the middle-aged patient and in 16.0% of the older patients and only in patients from the TRIAGE III study, representing a small subgroup of the total cohort, which warrants further internal and external validation of the predictive ability before it can be considered for use in the ED. Importantly, our results demonstrate the instrumental value of employing vital signs as part of risk assessment at triage, which previously have been demonstrated to predict mortality, [15, 41] but the predictive value is not equal across age groups. Vital signs have previously shown to be less predictive in the older prior to cardiac arrest and at triage [11, 42]. Additionally, vital signs have increased sensitivity when observed as serial measurements and might provide the best information with an individualised reference range, [10] however, this is not viable in the ED. Constructing age-dependent reference ranges or cut-offs for use in triage would be an alternative solution. We also demonstrate that use of routine biomarkers in risk assessment models could improve discriminative ability, but transference of these models directly into clinical use remains complicated. The performance of the investigated models in this study is comparable to other risk assessment tools of mortality assessed in a systematic review of scores based on different composition of vital signs and biomarkers [43]. In the cohort of middle-aged patients, our models of vital signs and biomarkers were comparable to the best performing tools in the review, which both used age and incorporated either vital signs or biomarkers.

Current triage algorithms have been validated to a limited extent in older patients, [3] and there is little or no evidence regarding performance in older patients or using age-adjustment among the most commonly used triage algorithms [12]. Only the Emergency Severity Index (ESI) algorithm has been investigated with regard to older patients, results were however conflicting [44–46]. Interestingly, the inclusion of age has been proposed in different track-and-trigger systems and predictions models, based on a perceived augmentation of predictive accuracy [47–51]. Furthermore, the physiological scoring systems, Simplified Acute Physiology Score (SAPS II) [52] and Acute Physiology And Chronic Health Evaluation (APACHE) [51], which are used for risk stratification of severity and risk of mortality in intensive care, include age. Finally, important factors affecting outcome for older patients are functional and cognitive status and comorbidity [53]. Incorporating these factors may increase the accuracy of the triage algorithm and lead to better management and treatment of older patients.

In summary, risk assessment can be strengthened using one of the described models, however, modifications for patients over 70 years of age should be considered. Our results indicate that incorporating age may improve the accuracy of risk assessment in older patients in future triage, but factors like functional and cognitive status should also be considered. Furthermore, new models should be validated in external cohorts before implementation. The experimental biomarker suPAR shows promise in the current study, but the results can only be considered as hypothesis-generation. Further research in this biomarker, the potential use, and possible interventions to elevated levels is warranted. Whether a new or redesigned triage algorithm will lead to better management and prognosis of older patients will require further prospective interventional studies.

### Strengths and limitations

This retrospective study combined two large prospectively collected cohorts from interventional trials conducted at two hospitals with inclusion of a representative and unselected cohort presenting around the clock, all days of the week, which is a major strength of the study. This study has several limitations. Patients with missing data were not included in the analyses, potentially causing a selection bias within the different risk assessment models investigated (i.e. patients with missing biomarkers (no blood analysis done) might be healthier than patients who had blood tests). Furthermore, although the missing data might not affect the comparison between age groups, the large difference in missing data (ADAPT missing in 50.3%, CTA: 65.9%, vital signs; 43.1%, biomarkers: 38.7%, and suPAR is missing in 85.0% of the total cohort) in the investigated models makes comparison of the risk assessment models difficult and should be interpreted with caution. Therefore, these results are only suitable for hypothesis generation. Measurement of vital signs are associated with some uncertainty, the temperature was measured using an ear thermometer, which might lead to underestimation of the actual temperature. All vital signs were recorded by hand and later entered manually in the database, which increases the risk of errors. The biomarker-based model in our study is based on the actual measurement value of the individual biomarkers, and this is not easy to implement in the clinical setting. Additionally, it is currently unknown whether they will have an impact when used as prognostic tools. This is also the case, regarding the experimental biomarker suPAR. This nonspecific marker of immune activation have previously demonstrated strong prognostic abilities, but only one trial has prospectively studied suPAR as a risk stratification tool and found no effect on mortality [26]. The suPAR level was only available in a small proportion of the total cohort and analysis of suPAR is not routinely performed in the ED, making it unavailable in most places or associated with increased costs. The investigated patients represent a subgroup of ED patients and the results might not be transferrable to different settings or in patients without need for blood sampling. Further research in the use this biomarker is required, before it can be considered for routine clinical use. The accuracy of the investigated triage algorithms might be artificially lower in the analyses, as a well-performing triage possibly translates to faster treatment of risk patients, hence lowering the risk of mortality. Furthermore, when the triage categories are used in the logistic models with only four levels, there is a limitation on the calculated AUC. Finally, we have not included comorbidity or diagnoses in our analyses, which would allow further stratification and precision in the models.

## Conclusion

The predictive ability of mortality in acutely admitted patients was lower in older patients compared to middle-aged patients in all investigated models, therefore modifications for age should be considered when constructing risk assessment model for use in the emergency department. Models based on vital signs or routine biomarkers provided the best models for prediction of 7-day mortality.

## Additional file

Additional file 1: **Table S1.** Comparison of Baseline characteristics of the TRIAGE II study and TRIAGE III study. Patients above 40 years were included in the current study **Table S2.** Comparison of AUCs of individual predictors in discriminating short-term mortality of ED patients, grouped according to age: 40–69 years (middle-aged), and 70+ years (older). **Figure S1.** Area under the Curve (AUC) for Receiver operating characteristics for all-cause mortality within 7 days for acutely admitted patients. Comparison of patients aged 40-69 (Middle-aged, blue colour), and patients aged 70+ (Older, red colour). The graph presents four different approaches of risk assessment of patients acutely presenting at the emergency department. Two different triage algorithms; Adaptive Process Triage (ADAPT) and Copenhagen Triage Algorithm (CTA), a predictive model using four vital signs (heart rate, arterial oxygen saturation, respiratory rate and systolic blood pressure), and a predictive model using levels of seven routine biomarkers (albumin, creatinine, c-reactive protein, haemoglobin, leucocytes, potassium, sodium). (DOCX 241 kb)

## Abbreviations

ADAPT: Adaptive Process Triage
AUC: Area under the curve
CI: Confidence interval
CRP: C-reactive protein
CTA: Copenhagen Triage algorithm
ED: Emergency department
suPAR: Soluble urokinase plasminogen activator receptor
