# Supplementary material for: Risk assessment models for potential use in the emergency department have lower predictive ability in older patients compared to the middle-aged for short-term mortality – a retrospective cohort study
Source: BMC Geriatr. 2019 May 16;19:134. doi: 10.1186/s12877-019-1154-7 (PMC6521424; doi:10.1186/s12877-019-1154-7)
Supplement: Supplementary file 1 — Table S1. Comparison of Baseline characteristics of the TRIAGE II study and TRIAGE III study. Patients above 40 years were included in the current study Table S2. Comparison of AUCs of individual predictors in discriminating short-term mortality of ED patients, grouped according to age: 40–69 years (middle-aged), and 70+ years (older). Figure S1. Area under the Curve (AUC) for Receiver operating characteristics for all-cause mortality within 7 days for acutely admitted patients. Comparison of patients aged 40-69 (Middle-aged, blue colour), and patients aged 70+ (Older, red colour). The graph presents four different approaches of risk assessment of patients acutely presenting at the emergency department. Two different triage algorithms; Adaptive Process Triage (ADAPT) and Copenhagen Triage Algorithm (CTA), a predictive model using four vital signs (heart rate, arterial oxygen saturation, respiratory rate and systolic blood pressure), and a predictive model using levels of seven routine biomarkers (albumin, creatinine, c-reactive protein, haemoglobin, leucocytes, potassium, sodium). (DOCX 241 kb) [file 12877_2019_1154_MOESM1_ESM.docx]

**Additional file 1**

Table S1. Comparison of Baseline characteristics of the TRIAGE II study and TRIAGE III study. Patients above 40 years were included in the current study

|  | TRIAGE II  N= 28,948 | TRIAGE III  N= 13,594 | P |
| --- | --- | --- | --- |
| Female sex, n (%) | 14,805 (51.1) | 7,075 (52.0) | 0.08 |
| Age (years), mean (SD) | 66.8 (15) | 68.3 (7.4) | 0.62 |
| Patients above 70 years (%) | 13,096 (45.2) | 6,793 (50.0) | 0.80 |
| **Biomarker levels, n (%)** | 13,909 (48.0) | 12,162 (89.5) |  |
| Albumin (g/L), median (IQR) | 40 (37–43) | 38 (34–42) | <0.001 |
| Creatinine (µmol/L), median (IQR) | 76 (62–97) | 77 (63–97) | <0.001 |
| CRP (mg/L), median (IQR) | 6 (3–49) | 6 (3–47) | <0.001 |
| Haemoglobin (mmol/L), median (IQR) | 8.3 (7.4–9.0) | 8.3 (7.4–9.0) | 0.57 |
| Leucocytes (× 10^9^/L), median (IQR) | 8.8 (6.7–11.8) | 8.6 (6.7–11.2) | <0.001 |
| Potassium (mmol/L), median (IQR) | 4.0 (3.7–4.3) | 4.0 (3.7–4.2) | <0.001 |
| Sodium (mmol/L), median (IQR) | 138 (135–141) | 139 (136–141) | <0.001 |
| Platelets (× 10^9^/L), median (IQR) | 241 (194–300) | 249 (201–309) | <0.001 |
| **Mortality** |  |  |  |
| Mortality within 2-days, (%) | 244 (0.8) | 92 (0.7) | 0.08 |
| Mortality within 7-days, (%) | 489 (1.7) | 246 (1.8) | 0.38 |
| CRP: C-reactive protein, IQR: Interquartile range, SD: Standard deviation | | | |

F

**Table S2. Comparison of AUCs of individual predictors in discriminating short-term mortality of ED patients, grouped according to age: 40-69 years (middle-aged), and 70+ years (older)**

| AUC, 95 % CI | Middle-aged N=22,653 | Older N=19,889 | P |
| --- | --- | --- | --- |
| Seven-day mortality | | | |
| Heart rate | 0.74 (0.67–0.81) | 0.61 (0.58–0.65) | <0.001 |
| Arterial oxygen saturation | 0.67 (0.59–0.74) | 0.64 (0.60–0.67) | 0.48 |
| Respiratory rate | 0.76 (0.70–0.82) | 0.68 (0.65–0.71) | 0.03 |
| Systolic blood pressure | 0.73 (0.67–0.79) | 0.68 (0.65–0.71) | 0.15 |
| Temperature | 0.52 (0.45–0.59) | 0.53 (0.49–0.56) | 0.93 |
| Albumin | 0.81 (0.77–0.85) | 0.69 (0.67–0.72) | <0.001 |
| Creatinine | 0.64 (0.57–0.70) | 0.66 (0.64–0.69) | 0.49 |
| CRP | 0.79 (0.76–0.83) | 0.69 (0.66–0.71) | <0.001 |
| Haemoglobin | 0.72 (0.66–0.77) | 0.59 (0.56–0.61) | <0.001 |
| Leucocytes | 0.67 (0.61–0.73) | 0.67 (0.64–0.70) | 0.87 |
| Potassium | 0.56 (0.49–0.63) | 0.60 (0.57–0.63) | 0.32 |
| Sodium | 0.64 (0.57–0.70) | 0.52 (0.49–0.55) | <0.001 |
| Platelets | 0.53 (0.46–0.60) | 0.52 (0.48–0.55) | 0.67 |
| suPAR | 0.82 (0.73–0.91) | 0.77 (0.72–0.82) | 0.32 |
| Two-day mortality | | | |
| Heart rate | 0.73 (0.65–0.82) | 0.62 (0.57–0.67) | 0.02 |
| Arterial oxygen saturation | 0.65 (0.55–0.74) | 0.66 (0.61–0.71) | 0.80 |
| Respiratory rate | 0.81 (0.73–0.89) | 0.72 (0.68–0.77) | 0.05 |
| Systolic blood pressure | 0.71 (0.63–0.79) | 0.72 (0.68–0.77) | 0.92 |
| Temperature | 0.50 (0.40–0.59) | 0.72 (0.68–0.77) | 0.39 |
| Albumin | 0.80 (0.74–0.85) | 0.68 (0.64–0.72) | <0.001 |
| Creatinine | 0.66 (0.57–0.74) | 0.70 (0.66–0.74) | 0.39 |
| CRP | 0.80 (0.75–0.86) | 0.68 (0.65–0.72) | <0.001 |
| Haemoglobin | 0.67 (0.58–0.75) | 0.56 (0.51–0.61) | 0.03 |
| Leucocytes | 0.65 (0.56–0.74) | 0.68 (0.64–0.72) | 0.60 |
| Potassium | 0.58 (0.49–0.68) | 0.64 (0.60–0.69) | 0.27 |
| Sodium | 0.64 (0.55–0.73) | 0.52 (0.47–0.56) | 0.01 |
| Platelets | 0.51 (0.42–0.61) | 0.50 (0.46–0.55) | 0.84 |
| suPAR | 0.82 (0.66–0.97) | 0.73 (0.64–0.81) | 0.32 |
| AUC: Area under the curve, CI: confidence interval, CRP: C-reactive protein, suPAR: soluble urokinase plasminogen activator receptor | | | |

**Figure S1.** Area under the Curve (AUC) for Receiver operating characteristics for all-cause mortality within seven days for acutely admitted patients. Comparison of patients aged 40-69 (Middle-aged, blue colour), and patients aged 70+ (Older, red colour). The graph presents four different approaches of risk assessment of patients acutely presenting at the emergency department. Two different triage algorithms; Adaptive Process Triage (ADAPT) and Copenhagen Triage Algorithm (CTA), a predictive model using four vital signs (heart rate, arterial oxygen saturation, respiratory rate and systolic blood pressure), and a predictive model using levels of seven routine biomarkers (albumin, creatinine, c-reactive protein, haemoglobin, leucocytes, potassium, sodium) **
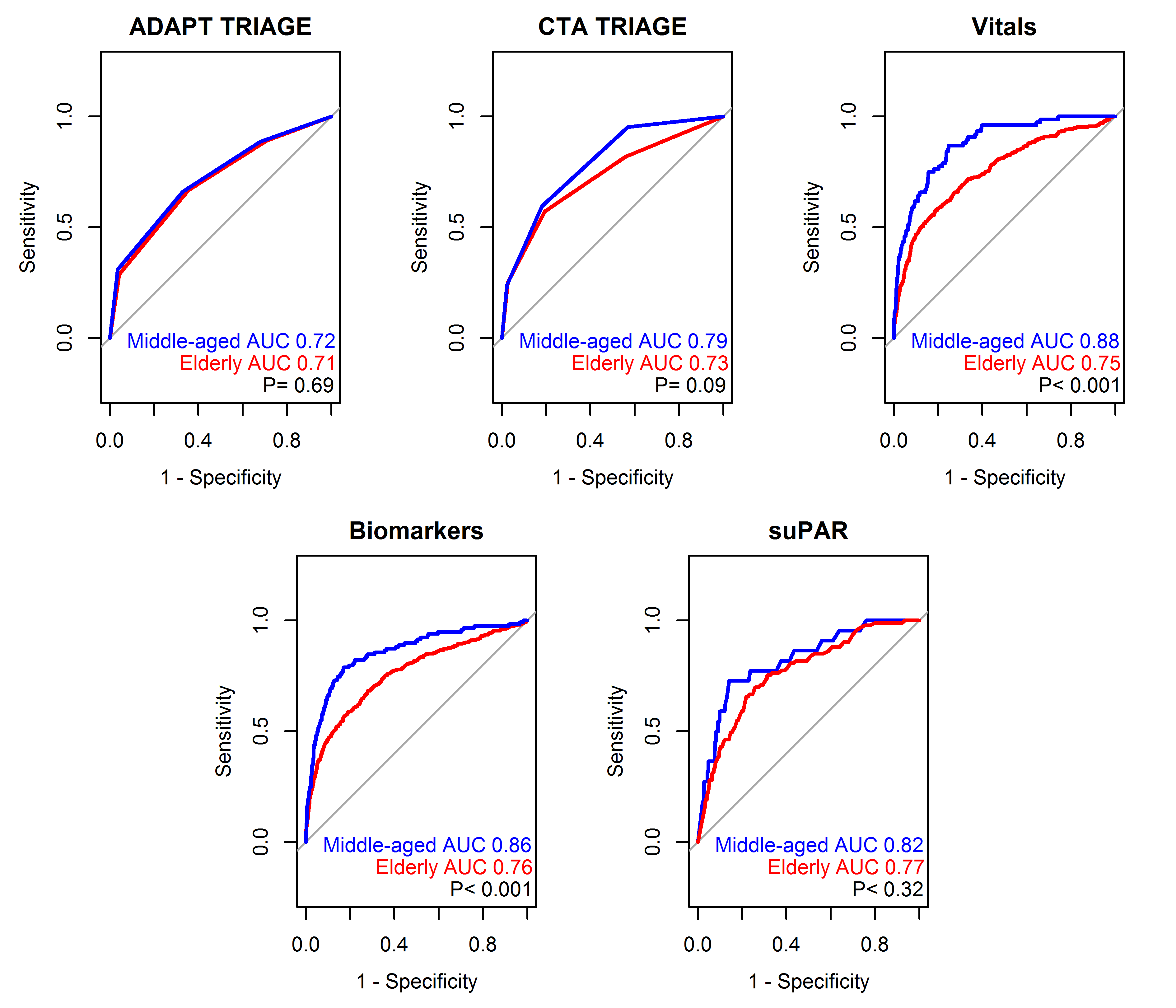
**
